# Cx43 phosphorylation on S279/282 and intercellular communication are regulated by IP_3_/IP_3_ receptor signaling

**DOI:** 10.1186/s12964-014-0058-6

**Published:** 2014-09-28

**Authors:** Man Kang, Na Lin, Chen Li, Qingli Meng, Yuanyuan Zheng, Xinxin Yan, Jianxin Deng, Yang Ou, Chao Zhang, Junqi He, Dali Luo

**Affiliations:** Department of Pharmacology, Capital Medical University, Beijing, 100069 China; Institute of Molecular Medicine, Peking University, Beijing, 100871 China; Department of Biochemistry, Capital Medical University, Beijing, 100069 China; Present address: National Institute for Radiological Protection, China CDC, Beijing, 100088 China

**Keywords:** Inositol 1,4,5-trisphosphate receptor, Gap junction, Connexin 43, Serine 279/282 phosphorylation, Intercellular communication, Ventricular myocyte

## Abstract

**Background:**

Inositol 1,4,5-trisphosphate receptor (IP_3_R) plays a pivotal role in the Ca^2+^ release process in a variety of cell types. Additionally, IP_3_R is distributed in ventricular intercalated discs, but its function(s) in this particular site remains unknown. Connexin (Cx43), the predominant gap junction (GJ) protein in ventricular myocardium, is linked to several signaling pathways that regulate Cx43 properties by (de)phosphorylation on multiple residues. Here, we investigated the regulatory role of IP_3_R in cell-cell communication and the mechanism(s) underlying this effect.

**Results:**

In neonatal rat and adult mouse ventricular myocytes IP_3_R co-localized and co-immunoprecipitated with Cx43 in GJ plaques detected by immunostaining and western blot assays. Blocking IP_3_R with antagonists or silencing pan-IP_3_R expression with shRNA hindered the 6-carboxyfluorescein (6-CFDA) diffusion through GJs and desynchronized Ca^2+^ transients among confluent neonatal myocytes in culture, whereas stimulation of IP_3_R with IP_3_ ester or ATP exerted the opposite effect. Likewise, 6-CFDA propagation through GJs was modulated by IP_3_R activation or inhibition in cell pairs of isolated adult cardiomyocytes. Furthermore, IP_3_R activation or IP_3_R suppression promoted or suppressed, respectively, Cx43 phosphorylation on S279/282. Site-directed mutagenesis indicated that expression of a mutant Cx43-S282A (alanine) inhibited S279/282 phosphorylation and GJ permeability, while the S279A mutant showed the opposite effect in ventricular myocytes. Expression of these mutants in HEK293 cells revealed that cells with a dual S279/282 mutation failed to express exogenous Cx43, whereas cells with a single S279 or S282 mutation displayed Cx43 overexpression with increased phosphorylation of S279/282 and promotion of intercellular communication.

**Conclusions:**

These results demonstrated, for the first time, that IP_3_R physically interacts with Cx43 and participates in the regulation of Cx43 phosphorylation on S279/282, thereby affecting GJ intercellular communication in ventricular myocytes.

**Electronic supplementary material:**

The online version of this article (doi:10.1186/s12964-014-0058-6) contains supplementary material, which is available to authorized users.

## Background

In heart, gap junctions (GJs) serve as intercellular communication channels to confer direct ion exchange and synchronization of electrical excitation between adjacent myocytes, thus allowing rhythmic coordinated myocardium contraction. In addition, they permit intercellular exchange of metabolites and small signaling molecules including cAMP, inositol 1,4,5-trisphosphate (IP_3_), and ATP, to maintain cellular homeostasis and couple biological activities between cells in the myocardium [1,2]. Abnormalities in GJs promote cardiac arrhythmias and apoptosis, two major complicating features of multiple cardiac pathologies [2-5]. Furthermore, mutations in the *connexin* gene, the main protein constituent of GJs, are linked to various human diseases, including cardiovascular anomalies [6-8]. Physiologically, connexin43 (Cx43), the predominant GJ protein in ventricular myocardium, is phosphorylated on multiple residues, a process that regulates its properties including assembling, trafficking, degradation, and electrical and metabolic coupling [1,2,9-12]. A variety of kinases, including protein kinase C (PKC), mitogen-activated protein kinase (MAPK), and Src, and connexin partners, such as ZO-1 and tublin, can alter Cx43 phosphorylation as well as its properties, and thereby affect heart function [10-14]. Of interest is phosphorylation of S262, S368, S279, and S282, which has been identified to link with the PKC, MAPK and PKA pathways, however, the precise role of a single kinase or Cx43 protein partner in the regulation of Cx43 phosphorylation is far from clear, because a kinase can phosphorylate more than one site and one site can be phosphorylated by various kinases and signaling pathways at the same time [12-15]. Thus, combined approaches including the use of Cx43 phosphorylation site-specific antibodies and alanine or aspartic acid (or glutamic acid) substitution as a silencer or a mimetic for phosphorylation can overcome these problems.

IP_3_ receptor (IP_3_R) protein, a family of three highly conserved isoforms, is expressed ubiquitously in the endoplasmic reticulum (ER) and plays a pivotal role in controlling the intracellular Ca^2+^ mobilization in non-electrical excitable cells. Though the three isoforms are found in the mammalian atrium and ventricles, they do not appear to be essential for cardiac excitation-contraction coupling [16-19]. However, studies have demonstrated that IP_3_R plays a critical role in regulating the local Ca^2+^ activity, including the nuclear Ca^2+^ signaling and affects gene transcription by the nuclear envelop-tethered IP_3_R-2 [17,18]. This receptor protein is also found in GJs in the ventricular myocardium [20], myoendothelium and sciatic nerve nodes [21,22], but its function in this particular site is not clear, yet. It is noteworthy that both ATP and IP_3_ can promote intercellular communication in cardiomyocytes [23,24] and non-myocytes [22,25-27]. Additionally, IP_3_R localizes to myoendothelium GJs on the endothelial cell side, but not on the vascular smooth-muscle cell side, leading to selective modulation of GJ coupling on the endothelial cell side by IP_3_ [21,27], suggesting that local IP_3_R is necessary for IP_3_-mediated GJ coupling.

Here, we investigated the potential contribution of GJ IP_3_R to the regulation of Cx43-associated intercellular communication, and the possible mechanism of this effect in ventricular cardiomyocytes. Because coordinated Ca^2+^ transients among connected cells reflect ion (electric) propagation through GJs [28,29], functional evaluation of cell-cell communication was carried out using synchronized Ca^2+^ transients and dye diffusion through GJs among connecting neonatal rat ventricular myocytes (NRVMs) in culture, an ideal native and non-invasive model for intercellular electrical and metabolic exchange [9,29]. Furthermore, using constructed site-directed mutagenesis we mutated Cx43 phosphorylation sites, to investigate target(s) that is likely involved in the IP_3_R-associated signal transduction.

## Results

### Structural association of IP_3_R with Cx43 in gap junctions of ventricular myocytes

**Figure 1:**
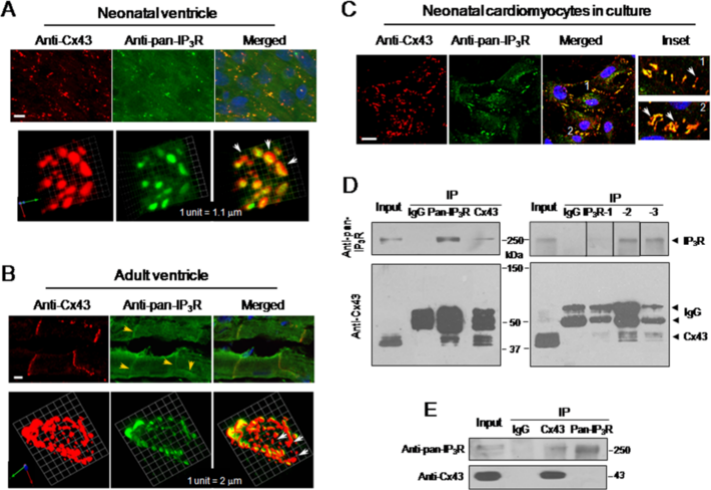
**Co-localization of IP**_**3**_ **R with Cx43 in gap junctions of ventricular myocytes.** Neonatal rat **(A)** and adult mouse ventricle tissues **(B)**, and cultured NRVMs **(C)** were co-immunostained with anti-Cx43 and anti-pan-IP_3_R antibodies. Representative confocal images show the subcellular distributions of Cx43 (red), pan-IP_3_R (green) and their co-localization (yellow) in the interfaces between adjacent myocytes. Three-dimensional reconstructions of a single disc of the end-to-end cells display that IP_3_R partially co-localized with Cx43 in GJs in ventricles (bottom panels in **A** and **B**). The enlarged interfaces 1 and 2 (inset in **C**) show the overlapped distribution of IP_3_R and Cx43 in NRVMs. Nucleus was stained with Hoechst 33258 (1 μg/ml). Scale bar: 10 μm. The yellow and white arrows in all panels indicate the enhanced IP_3_R signal in ventricles, and a fraction of Cx43 that is not associated with IP_3_R in GJs of connected NRVMs, respectively. Solubilized lysates from homogenized NRVMs **(D)** and mouse ventricles **(E)** were subjected to immunoprecipitation with anti-Cx43, anti-pan-IP_3_R, or anti-IP_3_R isoform antibodies as indicated. Representative western blots show the co-immunoprecipitated Cx43 or IP_3_R probed with anti-Cx43 or anti-pan-IP_3_R antibody. Data in all panels are representatives of 3–5 independent experiments.

To detect the three IP_3_R isoforms that are expressed in neonatal and adult ventricles [16-18], anti-pan-IP_3_R antibodies and anti-Cx43 antibodies were used to co-immunolabel samples. Figure 1A and B show representative interface region (upper panels) and three-dimensional reconstructions of the end-to-end intercalated discs between paired adjoining cardiomyocytes (bottom panels). Obvious co-localization of IP_3_R and Cx43 in GJ plaques (yellow color in upper panels) was observed in neonatal rat (Figure 1A) and adult mouse ventricular tissues (Figure 1B: here we used adult mice, because the IP_3_R distribution at the discs has already been reported in adult rat ventricles [20]). The front face disc view of GJ complexes demonstrated that the neonatal ventricular discs were fewer and smaller in size than those in adult discs, and the IP_3_R partially co-localized with Cx43, in particular in the larger GJs in both neonatal and adult ventricles (bottom panels). To conveniently expressing exogenous Cx43, NRVMs were used and co-immunostained with anti-pan-IP_3_R and anti-Cx43 antibodies. Similar to the neonatal tissue samples, IP_3_R clearly co-localized with Cx43, but there was still a small fraction of Cx43 that was not associated with IP_3_R in the GJ plaques of tissues and NRVMs (indicated with white arrows in Figure 1A and C). Additionally, it appears that there was more co-distribution of the two proteins in the total GJs of the neonatal samples than those in the adult samples, a difference likely due to a reduced IP_3_R expression after maturation [17-19].

Furthermore, a co-immunoprecipitation assay demonstrated that IP_3_R was detected in the Cx43 co-immunoprecipitated complex, and Cx43 (three bands around 41–45 kDa) was detectable in the reverse co-immunoprecipitation complexes from NRVMs by antibodies against pan-IP_3_R, or anti-IP_3_R-1, IP_3_R-2 or IP_3_R-3 isoforms (Figure 1D). In adult mouse ventricular samples, a detectable IP_3_R signal was also found in the Cx43 co-immunoprecipitated complexes, but Cx43 labeling was not detected in the complex co-immunoprecipitated respectively by two anti-pan-IP_3_R antibodies recognizing different section of amino acids (see Methods, Figure 1E). This partial difference, which is also consistent with the co-immunostaining assay (Figure 1A and B), between NRVMs and adult ventricular samples is likely because of lower IP_3_R expression and association with Cx43 in adult ventricles than in NRVMs.

### IP_3_R-associated regulation of intercellular chemical exchange in ventricular myocytes

**Figure 2:**
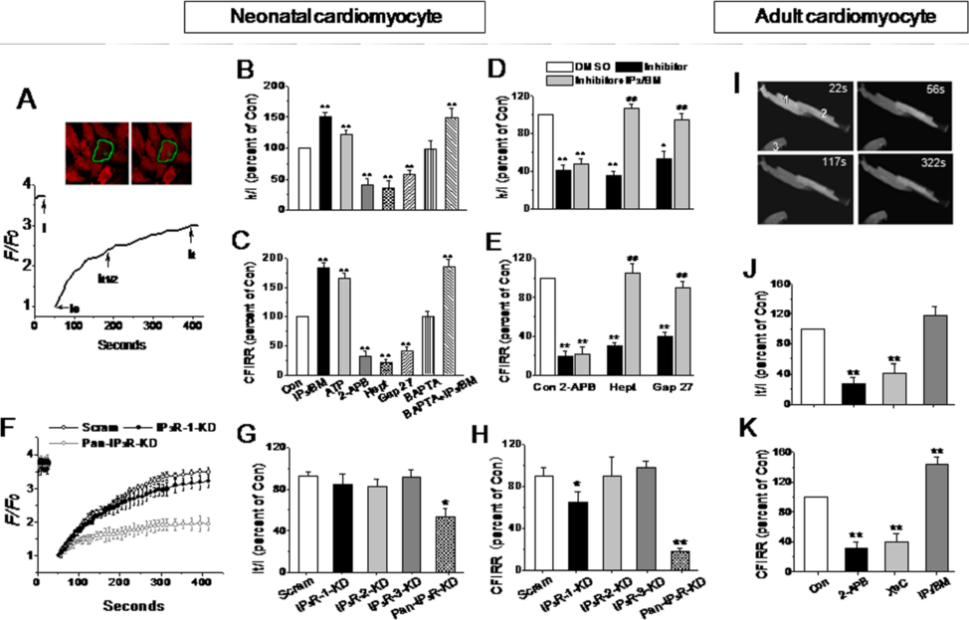
**IP**_**3**_ **R-associated regulation of the intercellular exchange in ventricular myocytes.** Representative images (inset) and traces of the bleached cell indicate 6-CFDA transfer through GJs from the connected NRVMs monitored by the FRAP method **(A)**. I, I0, It and It1/2 stand for the fluorescence intensity at the initial, after bleaching, recovery at 400 s, and at the time for 50% recovery, respectively. Effects of IP_3_/BM (20 μM, 6 minutes), ATP (5 μM, 3 minutes), 2-APB (3 μM, 10 minutes), heptanol (1 mM, 2 minutes), Gap 27 (300 μM, 30 minutes) and BAPTA/AM (200 μM, 10 minutes) on the fluorescence recovery in bleached cells are indicated with the percentage of two parameters; It/I **(B)** and CFIRR **(C)**. A rescue effect of IP_3_/BM was found on heptanol- or Gap 27-suppressed GJ, but not on 2-APB-inhibited GJ permeability **(D and E)**. Knockdown of pan-IP_3_R expression was obtained in NRVMs by shRNA against specific IP_3_R isoforms (Additional file 1: Figure S1). The traces of 6-CFDA recovery after bleaching **(F)** and their summarized data **(G and H)** between scramble control and IP_3_R deficient myocytes illustrate the effect of IP_3_R deficiency on the intercellular exchange. *P <0.05 and **P <0.01 vs*.* Con (vehicle) or scramble shRNA, and ^##^ *P* <0.01 vs*.* heptanol or Gap 27 treated cells, n = 7–25 independent experiments for each bar. Representative confocal images **(I)** of paired end-end cardiomyocytes isolated from mouse ventricles illustrate the fluorescence recovery in bleached cell 1 due to 6-CFDA diffusion through GJs from the connected cell (cell 2) monitored by FRAP method. Cell 3 is a background control. The statistical data depict the effects of IP_3_/BM (20 μM, 6 minutes), 2-APB (5 μM, 10 minutes) and XeC (10 μM, 20 minutes) in bleached cells **(J and K)**. **P <0.01 vs*.* Con (0.1% DMSO), n = 15–20 paired cells for each bar.

To explore the possible regulatory effect of IP_3_R on GJ coupling, the intercellular chemical exchange in response to the IP_3_R agonists, myo-inositol 1,4,5-trisphosphate hexakis (butyryloxymethyl) ester (IP_3_/BM) and ATP, and the IP_3_R antagonist, 2-aminoethoxydiphenyl borate (2-APB), was examined using the FRAP method [30-32]. For comparison, the GJ uncouplers, heptanol (1 mM) and Gap 27 (300 μM), a peptide that mimics short sequences in the extracellular loop 2 of Cx43 and inhibits GJs directly and specifically [33,34], were also used. In an NRVM monolayer loaded with 6-carboxyfluorescein diacetate (6-CFDA), a cell was selected and bleached with a strong laser light (Figure 2A, see Methods). The fluorescence recovery in the bleached cell because of free 6-CFDA diffusion through GJs rather than other routes from adjacent cells was calculated and the GJ permeability is represented by two characteristic values: the recovery degree (It/I) and the recovery speed {comparative fluorescence intensity recovery rate (CFIRR) = [(It − I0)/(I − I0) × 100%]/t} as reported with minor modifications [30-32]. Here, I, I0, It and It1/2 stand for the fluorescence intensity at the initial, after bleaching, recovery at 400 seconds and at the time for 50% recovery, respectively.

Like heptanol and Gap 27, 2-APB significantly suppressed the 6-CFDA recovery in the bleached NRVMs, whereas, IP_3_/BM, a membrane permeable IP_3_ analogue, and ATP augmented the dye diffusion (Figure 2B and C). Importantly, IP_3_/BM or ATP could restore the reduced GJ communication by heptanol or Gap 27, but not by 2-APB (Figure 2D and E). Chelating the intracellular Ca^2+^ with BAPTA/AM (200 μM, for 10 minutes) did not affect the dye propagation under the resting state or the promotion effect of IP_3_/BM. Thus, these data suggest that the mechanism of the IP_3_R-associated regulation of GJ intercellular communication is different from that of gap uncouplers, and this effect of IP_3_R is independent of the internal Ca^2+^ level.

To further determine the IP_3_R subtype responsible for this regulation of intercellular communication, we suppressed the expression of IP_3_R-1, IP_3_R-2 and IP_3_R-3 with shRNA. After cells were transduced with shRNA against each IP_3_R isoform for 48 hours, NRVMs exhibited approximately 70% inhibition of the expression of each IP_3_R isoform, and a 62% reduction in relative pan-IP_3_R abundance was observed when the three shRNAs were used together (Additional file 1: Figure S1). A significant reduction in fluorescence recovery was also observed in the bleached pan-IP_3_R-knockdown cells, but not in the IP_3_R isoform-knockdown cells, except for a detectable decrease in the recovery rate observed in IP_3_R-1 deficient cells (Figure 2F-H). This implies that all the three IP_3_R isoforms participate in the regulation on GJ permeability in NRVMs. Again, neither IP_3_/BM nor ATP could rescue the suppressed dye transfer through GJs in pan-IP_3_R-knockdwon cells.

Similarly to the neonatal cells, adult paired end-to-end cardiomyocytes exhibited an increased GJ exchange upon IP_3_R activation with IP_3_/BM (20 μM, for 10 minutes) and a decreased GP exchange upon IP_3_R blockade with either 2-APB (5 μM, for 10 minutes) or an another inhibitor of IP_3_R xestospongin (XeC 10 μM, for 20 minutes), as assessed by the FRAP method (Figure 2I-K).

### IP_3_R-associated regulation of intercellular electrical spreading in ventricular myocytes

**Figure 3:**
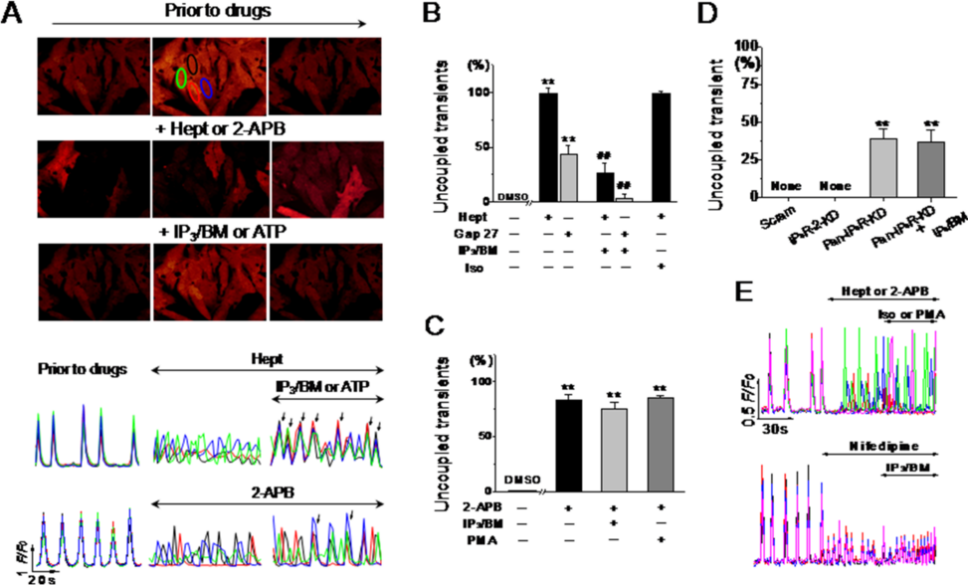
**IP**_**3**_ **R-associated regulation of electronic spreading among monolayer neonatal ventricular myocytes.** Coordinated spontaneous Ca^2+^ oscillations were measured by confocal microscopy in cultured NRVMs loaded with fluo-4. Representative images and traces illustrate the global Ca^2+^ transients prior to and after 2-APB (3 μM, 10 minutes) or heptanol (1 mM, 2 minutes) treatment followed by addition of IP_3_/BM (20 μM, 6 minutes) or ATP (5 μM, 3 minutes) **(A)**. Ca^2+^ transient uncoupling, represented by the percentage of dysynchronous transients in four to five connected cells indicated with circles in control image, were found in heptanol, 2-APB **(B and C)**, or pan-IP_3_R shRNA **(D)** treated cells. IP_3_/BM and ATP could rescue the uncoupled transients induced by heptanol but not by 2-APB or pan-IP_3_R shRNA. Nifedipine (0.3 μM, 10 minutes), PMA (1 μM, 20 minutes) or isoprenaline (Iso, 0.1 μM, 2 minutes) did not mimic the effect of 2-APB or IP_3_/BM on coupled spontaneous Ca^2+^ oscillations **(E)**. **P <0.01 vs*.* the cells treated with vehicle or scramble shRNA; ^##^ *P* <0.01 vs*.* heptanol alone, n = 10–23 independent determinations for each bar.

Next, the electrical spreading between adjacent cells was determined by assessing the coordination of spontaneous Ca^2+^ transients in monolayer NRVMs, because a direct linkage between GJs and coordinate Ca^2+^ transients have been identified [28,29]. Normally, all connected NRVMs oscillate simultaneously because of direct ion propagation through GJs, but this coupling property can be disrupted by GJ inhibition, causing desynchronized Ca^2+^ transients. Thus, the asynchronous Ca^2+^ oscillations among four to five adjacent cells represent a non-invasive interruption of electrical propagation through GJs, which is expressed as a percentage of the total transients during a 2-minute recording. As shown in Figure 3A-C, coordinated Ca^2+^ transients were discontinued by addition of the gap uncoupler, heptanol or Gap 27, and changed to asynchronous Ca^2+^ spiking. 2-APB (3 μM) that inhibited the 6-CFDA diffusion (Figure 2B) also interfered with the coordination rhythm of Ca^2+^ transients. Similarly, knocking down pan-IP_3_R expression, but not any IP_3_R isoform, with shRNA caused partial desynchronization of the rhythmic Ca^2+^ oscillations. Moreover, consistent with the FRAP assay results, IP_3_/BM and ATP resynchronized these distorted Ca^2+^ transients in heptanol- or Gap 27-treated cells (Figure 3B and Additional file 2: Video 1), but not in 2-APB- (Figure 3C and Additional file 3: Video 2) or pan-IP_3_R shRNA-treated cells (Figure 3D). Neither isoprenaline, which stimulates the β_1_-adrenergic receptor, nor phorbol myristate acetate (PMA), which activates PKC, mimicked this recovery effect of IP_3_/BM. Furthermore, nifedipine, which inhibits L-type Ca^2+^ channels, did not elicit any uncoupling effect on Ca^2+^ oscillations (Figure 3B,C and E), although all of these reagents did affect the Ca^2+^ signaling rate and amplitude (Figure 3E, data not completely shown).

### IP_3_R-associated regulation of Cx43 phosphorylation in ventricular myocytes

**Figure 4:**
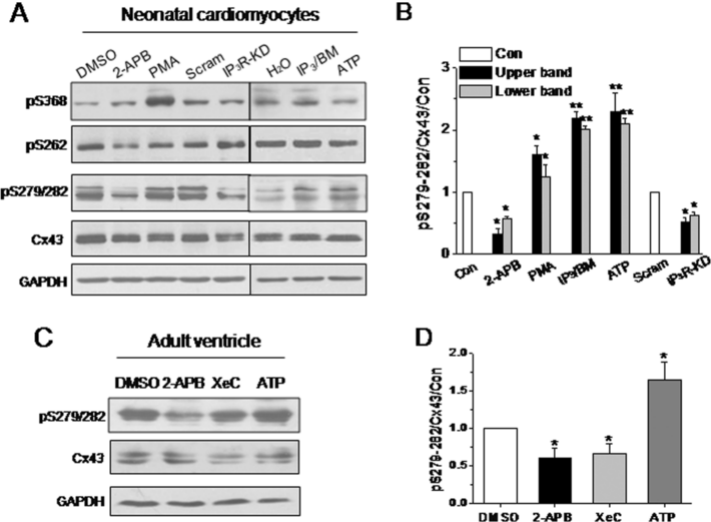
**IP**_**3**_ **R-associated regulation of Cx43 phosphorylation in ventricular cardiomyocytes.** Solubilized lysates from homogenized NRVMs **(A)** and mouse ventricles **(C)** that had been treated with different reagents as indicated were subjected to specific anti-phosphor Cx43 and anti-Cx43 antibodies, respectively. Representative western blots of pS368, pS262, pS279/282 and Cx43 as indicated **(A)** and their normalized relative abundances in NRVMs **(B)**, and the blots of pS279/282 and Cx43 **(C)** and their normalized data in adult ventricles **(D)** depict the effects of 2-APB, shRNA against pan-IP_3_R, IP_3_/BM and ATP at the concentrations similar to those in functional evaluation on Cx43 phosphorylation. *P <0.05 and **P < 0.01 vs*.* DMSO- or scramble shRNA-treated cells, n = 3–4 independent experiments for each bar.

Compared with other connexins, Cx43 has a larger intracellular C-terminal tail, and (de)phosphorylation of serines in this domain represents an important regulatory mechanism for GJ gating, assembly, trafficking, and degradation. To investigate whether IP_3_/IP_3_R pathway affects GJ permeability by mediating the Cx43 phosphorylation, the relative abundance of pS262, pS368, and pS279/282 was analyzed by western blot analysis using phospho-specific antibodies. While one band of about 43 kDa appeared with the pS262 or pS368 antibody labeling, two clear bands were detected with the pS279/282 antibody labeling of samples from both NRVMs and adult ventricles (Figure 4A). Activation of PKC with PMA caused a significant increase in S368 phosphorylation and a slight increase in S279/282 phosphorylation, which is consistent with previous reports [10,12]. However, interestingly, inhibition of IP_3_R with 2-APB (3 μM) or shRNA against pan-IP_3_R significantly suppressed the levels of S279/282 phosphorylation, while no significant change was observed in S262 or S368 phosphorylation (Figure 4A and B). Furthermore, activation of IP_3_R with IP_3_/BM (20 μM) or ATP (5 μM) promoted Cx43 phosphorylation at S279/282 in NRVMs, and similar results were obtained in adult ventricles that were retrogradely perfused in a Langendorff system with HEPES-buffered Tyrode solution containing 2-APB, XeC, or ATP for 10, 20, or 5 minutes, respectively (Figure 4C and D).

### Effects of S279 and S282 mutations on Cx43-assciated junction coupling in ventricular myocytes

**Figure 5:**
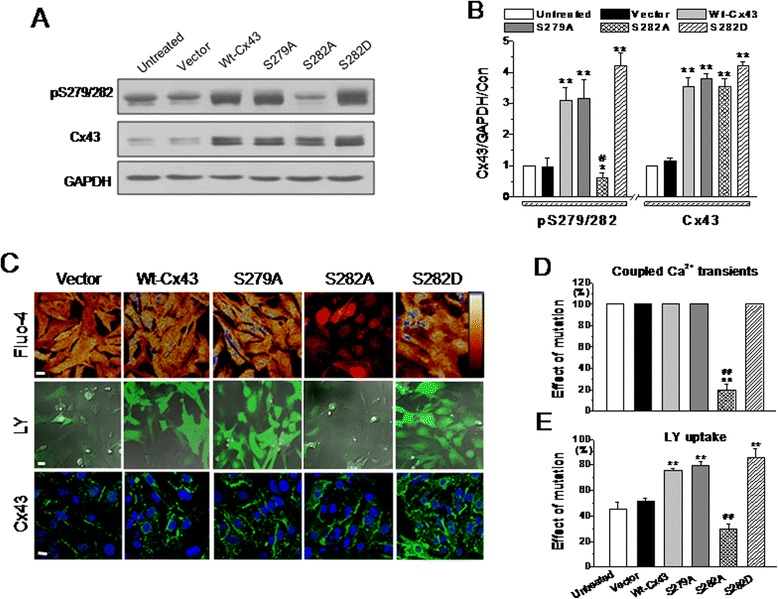
**Effects of S279 and S282 mutation on Cx43-associated function and expression in neonatal ventricular myocytes.** Cultured NRVMs were transduced with vector, wt-Cx43, S279A, S282A, or S282D mutant for 24 hours. S279/282 phosphorylation and Cx43 expression were detected by western blots **(A)**. The statistical data **(B)** depict the effect of mutating S279 or S282 to alanine or aspartic acid on the relative abundances of pS279/282 and Cx43 expression. These transduced cells were also loaded with fluo-4 or LY dye, or immunostained with anti-Cx43 antibody, and then analyzed by confocal microscopy. Representative images **(C)** and the summarized data from 4–6 independent experiments illustrate the changes in coupled Ca^2+^ transients **(D)**, LY uptake **(E)** and Cx43 distribution (bottom panels in C) because of *Cx43* gene manipulation as indicated. Scale bar: 10 μm. **P < 0.01 vs*.* vector cells; ^#^ *P* < 0.05 and ^##^ *P* < 0.01 vs*.* wt-Cx43 cells in all panels, n = 4–8 independent determinations for each bar.

**Figure 6:**
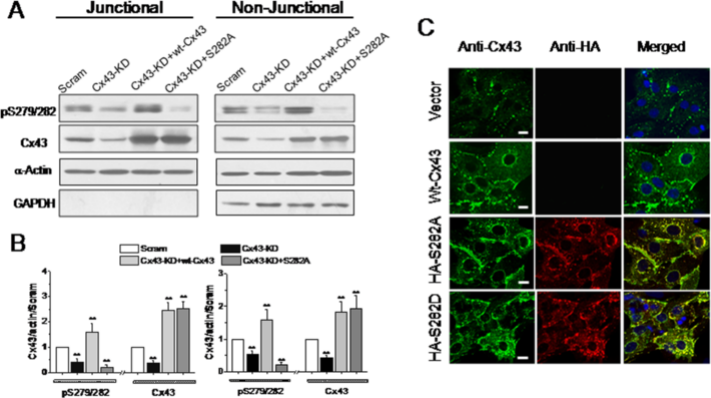
**Effect of S282 mutation on Cx43 expression in endogenous Cx43-knockdown neonatal ventricular myocytes.** NRVMs were transduced with scramble or siRNA to knockdown Cx43 (Cx43-KD, m.o.i. = 60) for 48 hours followed by further exposure of wt-Cx43 (m.o.i = 20) or S282A mutant (m.o.i. = 2) for 24 hours. The pS279/282 levels in triton X-100 soluble and insoluble fractions was determined by western blot **(A)**, and normalized by the level of actin and then the level of Cx43 in scramble cells **(B)**. The detection of GAPDH observed in non-junctional but not in junctional fraction represents a successful separation of non-junctional and junctional Cx43. N = 4 independent determinations for each bar, and ** denotes *P* < 0.01 vs. scramble cells. Cultured NRVMs were co-immunostained with anti-Cx43 and anti-HA-probe antibodies after transduced with scramble, wt-Cx43 (m.o.i = 20), S282A-HA (m.o.i = 2), or S282D-HA mutant (m.o.i = 2) for 24 hours **(C)**. Representative confocal images show the subcellular distributions of total Cx43 (green), exogenous Cx43-HA (red) and their co-localizations in S282D-HA and S282A-HA cells (yellow). Scale bar: 10 μm.

To elucidate whether S279/282 phosphorylation affects the gap permeability in cardiomyocytes, we used site-directed mutagenesis to mutate S279 and S282 into alanine or aspartic acid in rat *Cx43* gene (wt-Cx43). Compared with the vector control, transduction of adenovirus carrying S279A (5 m.o.i.), S282A (2 m.o.i.), S282D (2 m.o.i.), or wt-Cx43 (20 m.o.i.) into monolayer NRVMs generally caused similar increases in Cx43 expression and S279/282 phosphorylations in all groups of cells, except for a reduced S279/282 phosphorylation in S282A- (Figure 5A and B) and S279A/282A-transduced myocytes (data not shown). To avoid the infection efficiency differences among different groups, lucifer yellow (LY), a GJ-permeable but membrane-impermeable dye, combined with dextran-rhodamine B, a GJ- and membrane-impermeable dye, were used to evaluate the GJ permeability in monolayer NRVMs [35,36]. We found that all living cells were impermeable to dextran-rhodamine B, but permeable to LY with different efficiencies. Cells expressing the S279A mutant, S282D mutant, or wt-Cx43 exhibited intact synchronous Ca^2+^ oscillations and elevated LY uptake compared with vector-treated cells, whereas cells expressing the S282A mutant displayed uncoordinated Ca^2+^ spiking and decreased LY uptake (Figure 5C-E). Identical results were also found in the cells with double mutations of S279 and S282 (data not shown). Immunostaining assay with anti-Cx43 antibody confirmed that significantly more Cx43 was found on the cell membrane of all Cx43-modified cells compared with untreated control cells (bottom images in Figure 5C). To exclude the possibility that Cx43-S282A does not form GJ plaques [37], thereby causing suppression of the intercellular permeability compared with those in other Cx43-maniputated cells, the relative abundance of Cx43 expression in NRVMs was further determined by western blotting of triton-insoluble and -soluble fractions, a method used to distinguish between GJs and GJ precursors [13,38], after knockdown of endogenous Cx43 expression by specific siRNA [38]. There was no significant difference in both Cx43 fractions between the cells treated with wt-Cx43 or S282A (Figure 6A and B), but much lowered pS279/282 level in S282A cells than in wt-Cx43 cells, indicating that Cx43-S282A, like wt-Cx43, was able to dock in the GJ plaques in cardiomyocytes, but was unable to efficiently couple the intercellular communication. Furthermore, immunostaining assay with anti-HA antibody was used to confirm that exogenous Cx43 did distribute on the cell membrane of the Cx43-modified cells (Figure 6C).

**Figure 7:**
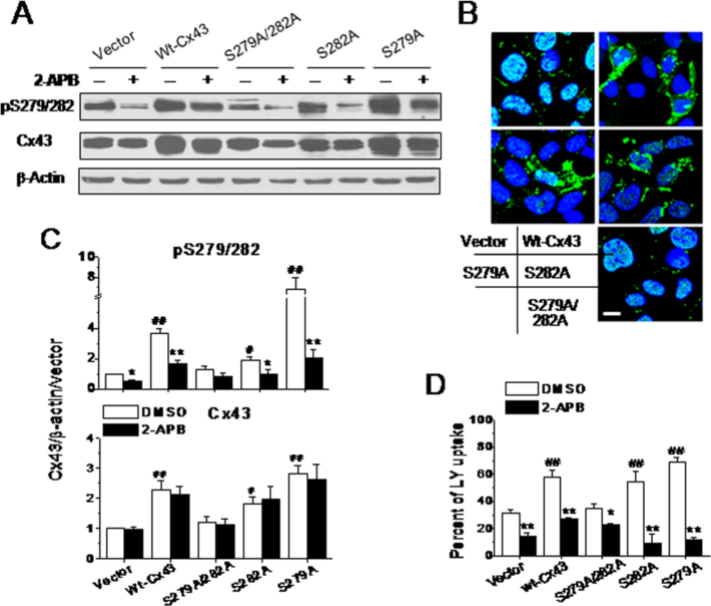
**Effects of S279 or S282 mutation on Cx43 expression and gap junction permeability in non-muscle cells.** Representative western blots of lysates from HEK293 cells **(A)**, which had been transduced with plasmids carrying rat nonspecific sequence (vector), wt-Cx43, S279A, S282A or S279A/282A genes for 48 hours, show the changes in the relative levels of pS279/282 and Cx43 expressions due to the mutations. Here, one blot membrane with 6 left bands and another membrane with 4 right bands from the same lysates were connected together to show all treatment groups. Some groups of the HEK293 cells were also treated with 5 μM 2-APB for 10 minutes, while others were stained with anti-Cx43 antibody to examine the subcellular distribution of Cx43 upon different treatments as indicated **(B)**. Scale bar: 10 μm. The statistical data normalized by β-actin depict the effects of different Cx43 mutants and 2-APB on S279/282 phosphorylation and Cx43 expression **(C)**. N = 4 independent determinations for western blotting and immunostaining tests, respectively. In addition, the statistical data of LY uptake depict the effects of the different mutants on GJ permeability **(D)**. N = 5–6 independent determinations for each bar. *P <0.05 and **P <0.01 vs*.* DMSO (0.1%); ^#^ *P* <0.05 and ^##^ *P* <0.01 vs*.* vector, respectively.

To further determine the role of S279/282 phosphorylation in the regulation of GJ permeability, HEK293 cells (they also possess 2-APB-sensitive endogenous Cx43 [39]) expressing the abovementioned mutants were used to determine the differences in exogenous Cx43 phosphorylation on S279/282 and in GJ permeability. Interestingly, only cells transduced with the S279A/282A mutant failed to increase the Cx43 expression, while all the other cells treated with wt-Cx43, S279A, or S282A mutant displayed elevated S279/282 phosphorylation and Cx43 expression, which was distributed in the cytosol and on the cell surface (Figure 7A-C). Consistently, functional evaluation of GJ permeability showed a linkage of LY uptake with the increased Cx43 phosphorylation on S279/282 and exogenous Cx43 expression, and that 5 μM 2-APB abrogated the S279/282 phosphorylation as well as the LY uptake in the control and all the Cx43-manipulated HEK293 cells (Figure 7D).

## Discussion

In this study, we demonstrated, for the first time, that the IP_3_R-associated signaling pathway regulates GJ intercellular communication by affecting Cx43 phosphorylation on S279/S282 in ventricular myocytes, and that the changes in the internal Ca^2+^ level are probably not involved.

Many studies have demonstrated that G-protein coupled receptor (GPCR) activators including noradrenaline, ATP and vasopressin can promote GJ intercellular communication in cardiomyocytes and non-myocytes [21-26], whereas IP_3_R inhibition by 2-APB interferes with the intrinsic cell-cell communication [38,39]. Therefore, IP_3_ and ATP due to their rapid free diffusion through GJ channels have been proposed as important molecules for synchronizing Ca^2+^ signaling between adjacent cells in GPCR pathway activation [22-25]. However, there was no information on whether the localized IP_3_R to GJs is involved in this regulation until it was reported that IP_3_R-1, which is selectively localized to the endothelial side of GJs, allows endothelial cells to respond to IP_3_ from smooth-muscle cells [21,27]. Our data are in good agreement with these findings. Deficiency in pan-IP_3_R expression led to reduced gap permeability and induction of uncoordinated Ca^2+^ transients in cardiomyocytes (Figures 2 and 3). More importantly, IP_3_/BM selectively restored the blocked GJ permeability and asynchronous Ca^2+^ transients by heptanol or Gap 27, but not by interference with IP_3_R by 2-APB or shRNA, indicating that this action of IP_3_-associated signaling occurs on the site of GJ plaques rather than on the sarcoplasmic reticulum stores. Additionally, the physical association between IP_3_R and Cx43 in the GJs and the alterations in Cx43 phosphorylation confer the possibilities of their molecular interactions directly or indirectly, and thus affecting the Cx43 function (Figure 1).

Cx43 has a large C-terminus domain in the cytosol, allowing it to interact with other proteins including kinases, phosphatases, membrane receptors, cell signaling and scaffolding proteins [9-15]. It is well recognized that phosphorylation of different Cx43 residues in the C-terminal leads to distinguishable changes in Cx43 properties including GJ gating, Cx43 assembly, trafficking, and degradation [1,13-15,37]. It has also been shown that Cx43 phosphorylation on one residue sometimes affects Cx43 interactions with other partner proteins or phosphorylation of another site [40,41]. S279 and S282 residues together with S255 and S262 have been identified as the recognition sites for the MAPK signaling pathway, which can promote their phosphorylations and induce a decreased conductivity of GJs in several cell lines [11,12,14]. However, the specific effect of the S279/282 sites on the acute regulation of GJ intercellular communication is rather sparse and obscure. It appears that MAPK is necessary but not sufficient to lead acute closure of GJ channels, and that other pathways including PKC activation are converged to regulate Cx43 coupling in response to various growth factors and v-Src activation [12,14]. However, Warn-Cramer, et al. have reported that activation of MAPK and the following phosphorylation of Cx43 at the site(s) of S279 and/or S282 was sufficient to disrupt GJ communication in Hela cells, a conclusion mainly based on that cells with S255A, S279A, and S282A Cx43 mutations failed to close GJs in response to MAPK activation, whereas the S255D Cx43 mutant established a level of GJ coupling comparable to that observed in cells expressing wt-Cx43 [11]. These conflict results reflect the complexity and diversity of Cx43 phosphorylation on multiple sites at same time upon activation of a signal pathway or a kinase, and thus diversified regulation of GJ function [1,15].

The data in this study demonstrated that a decrease in endogenous Cx43 phosphorylation on S279/282 down-regulated, whereas an increase in their phosphorylation up-regulated the basal GJ communication in cardiomyocytes (Figure 4). Exogenous expression of S279A, S282A or S282D in cardiomyocytes and HEK293 cells showed a close association between S279/282 phosphorylation and gap permeability (Figures 5 and 7). The accordant responses of Cx43 phosphorylation on S279 and S282 to IP_3_/IP_3_R signaling pathway and to mutation at S279 or S282 is specific by already ruling out some other possible interfering factors, such as intracellular Ca^2+^ change or non-specificity of the antibody, because 1) nifedipine which also altered internal Ca^2+^ signaling in NRVMs (Figure 3) did not affect Cx43 phosphorylation on S279/282 (data not shown); 2) S262 phosphorylation remained unaltered to changes in IP_3_R function status (Figure 4) or to S282A or S282D mutant treatment (data not shown); and 3) the detection of Cx43 in S282A-HA-treated cells with anti-HA antibody indicates that exogenous Cx43 was expressed in the site of GJs (Figure 6), thereby contribute for the distorted GJ function in these mutant-transduced cells (Figure 5). However, it is not clear about the ratio of exogenous Cx43 abundance among the total in one particular mutant, since it is hard to separate the exogenous protein from the endogenous protein in one blotting membrane simply by using anti-HA and anti-Cx43 antibodies. Therefore, these data here demonstrated a close link between IP_3_/IP_3_R pathway and Cx43 phosphorylation on S279/282, and also strongly suggest an important role of their phosphorylation in the regulation of Cx43-associated function.

Although S282A mutant, but not S279A mutant, suppressed both dye diffusion and coupled Ca^2+^ oscillations through GJs, it appears that failure to phosphorylate S282 also affected the phosphorylation of S279, because no further S279/282 phosphorylation above baseline was observed in S282A-treated cardiomyocytes (Figure 5), but an elevation in S279/282 phosphorylation and inhibitory effect of 2-APB on S279/282 phosphorylation and LY uptake were observed in S282A-treated-HEK293 cells (Figure 7). Additionally, S282A-mutated cardiomyocytes (Figure 5) showed exactly the same effects on LY uptake and Ca^2+^ coordination as those observed in S279/282-mutated cardiomyocytes (data not shown) and HEK293 cells (Figure 7). Thus, the site of S279 cannot be excluded from the regulatory role of S279/282 phosphorylation in GJ communication. It is also noticeable that unlike the observation in S282A-treated cardiomyocytes that did express the exogenous Cx43 (Figure 6), the expression of the rat Cx43-S279/282 mutant in HEK293 cells failed to induce additional Cx43 expression, but the other mutants did (Figure 7). A similar observation has been reported in ovarian granulosa, in which the S279/282 mutants were confined to intracellular sites, with few GJs [37]. However, a different effect of S279/282 phosphorylation has been demonstrated to disrupt GJ assembly by triggering endocytosis of Cx43 prior to its assembly in pancreatic cancer cells [42]. Nevertheless, all the results suggest the important role of S279/282 phosphorylation in the regulation of Cx43 assembly/expression in non-myocytes.

Therefore, this study showed that the IP_3_/IP_3_R pathway links with Cx43 phosphorylation on S279/282, providing a simple but rather rapid regulation for GJ coupling. In particular, this association is probably necessary for cardiac performance during sympathetic nervous activation of both α_1_- and β_1_-adrenergic receptors. The formation of IP_3_ and activation of GJ IP_3_R consequently induces a prompt elevation in intercellular coupling to match the accelerated electric triggering and myocardium contraction by β_1_-receptor stimulation. It is not clear, yet, how IP_3_R interacts with Cx43 and affects Cx43 phosphorylation on S279/282. As IP_3_/BM and the deficiency in IP_3_R expression efficiently induced obvious changes in Cx43 phosphorylation and in regulation of gap permeability, PKC and MAPK are probably not the signaling molecules in this regulation. Some studies have demonstrated that sodium channels also co-localize with Cx43 in GJs, but their function is unknown [43-45]. Therefore, further studies are needed to define how IP_3_R interacts with Cx43 and regulates its phosphorylation, which may provide a clue for interpreting how an ion channel interacts with connexins.

## Conclusions

Taken together, these observations demonstrate that IP_3_R regulates Cx43-associated intercellular communication in a manner independent of internal Ca^2+^ change, but rather reflecting a physiological role of IP_3_R interaction with Cx43, thereby regulating Cx43 phosphorylation on S279/282 in ventricular myocytes.

## Methods

This study was approved by the Capital Medical University Animal Care and Use Committee, and all studies were conducted in accordance with “Guide for the Care and Use of Laboratory Animals” adopted by the Beijing Government and “Guide for the Care and Use of Laboratory Animals” published by the US National Institutes of Health (publication No. 85–23, revised 1996).

The full Methods section refers to the Additional file 4.

## Additional files

Additional file 1: Figure S1 Evaluation of interfering IP_3_R isoform and pan-IP_3_R expression with shRNA. Representative western blots of knockdown of IP_3_R isoform or pan-IP_3_R in cultured NRVMs by shRNA against the distinctive IP_3_R isoform (A and B) and pan-IP_3_R (C). The relative abundances of each isoform IP_3_R and pan-IP_3_R were normalized by GAPDH and then scramble control. ***P* <0.01, n = 3–5 independent experiments for each panel. Additional file 2: Video1 The interrupted coordination in NRVM Ca^2+^ transients induced by heptanol (1 mM, 2 minutes) is recovered by ATP (5 μM, 2 minutes). Additional file 3: Video2 The interrupted coordination in NRVM Ca^2+^ transients induced by 2-APB (3 μM, 6 minutes) could not be rescued by ATP (5 μM, 2 minutes). Additional file 4
**Full Methods.**

## Abbreviations

IP_3_R: Inositol 1,4,5-trisphosphate receptor
LY: Lucifer yellow
Cx43: Connexin 43
NRVMs: Neonatal rat ventricular myocytes
FRAP: Fluorescence recovery after photobleach
HBSS: HEPES-buffered salt solution
XeC: Xestospongin C
2-APB: 2-aminoethoxydiphenyl borate
6-CFDA: 6-carboxyfluorescein diacetate
ER: Endoplasmic reticulum
SR: Sarcoplasmic reticulum
S279/282: Serines 279/282
S279A/282A: Serine 279 and serine 282 with alanine
S282D: Serine 282 with aspartic acid
GPCR: G-protein coupled receptor
GAPDH: Glyceraldehyde-3-phosphate dehydrogenase
