# Supplementary figures and images for: Cx43 phosphorylation on S279/282 and intercellular communication are regulated by IP3/IP3 receptor signaling
Source: Cell Commun Signal. 2014 Sep 28;12:58. doi: 10.1186/s12964-014-0058-6 (PMC4195880; doi:10.1186/s12964-014-0058-6)

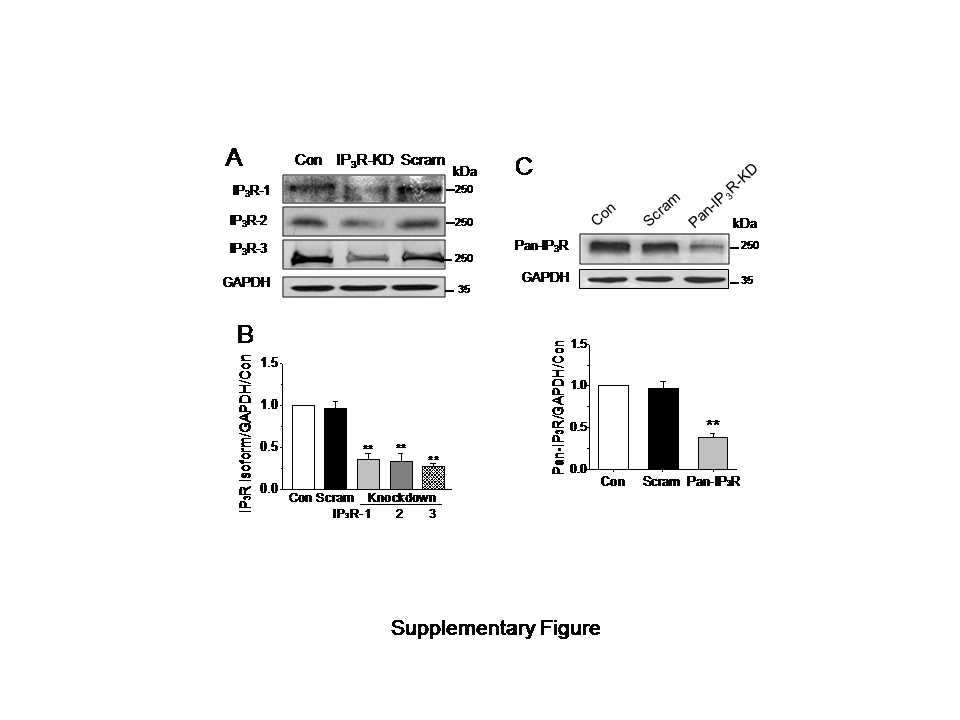

Supplement: Additional file 1: Figure S1 — Evaluation of interfering IP3R isoform and pan-IP3R expression with shRNA. Representative western blots of knockdown of IP3R isoform or pan-IP3R in cultured NRVMs by shRNA against the distinctive IP3R isoform (A and B) and pan-IP3R (C). The relative abundances of each isoform IP3R and pan-IP3R were normalized by GAPDH and then scramble control. **P <0.01, n = 3–5 independent experiments for each panel. [file 12964_2014_58_MOESM1_ESM.tiff]
