# Supplementary material for: Cx43 phosphorylation on S279/282 and intercellular communication are regulated by IP3/IP3 receptor signaling
Source: Cell Commun Signal. 2014 Sep 28;12:58. doi: 10.1186/s12964-014-0058-6 (PMC4195880; doi:10.1186/s12964-014-0058-6)
Supplement: Additional file 4 — Full Methods. [file 12964_2014_58_MOESM4_ESM.doc]

**Additional file**

**Methods**

***Materials***

Myo-inositol 1,4,5-trisphosphate hexakis (butyryloxymethyl) ester (IP_3_/BM, purity>95%) was synthesized as instructed [1]. This ester induced maximal Ca^2+^ release effect in Ca^2+^-free medium in HEK293 cells and potentiation of spontaneous Ca^2+^ oscillations in NRVMs after its addition of 6 min (data not shown), thereby all the data of IP_3_/BM effect here were collected after its addition of 6 min. Fluo-4/AM, 6-carboxyfluorescein diacetate (6-CFDA), Lucifer yellow (LY) and dextran-rhodamine B were obtained from Molecular Probes (Invitrogen Inc, Carlsbad, California, USA). Xestospongin C (XeC) and 2-aminoethoxydiphenyl borate (2-APB) were from Calbiochem (Merck Inc. Bad Soden, Schwodbach, Germany). An anti-pan-IP_3_R antibody, mapping between amino acids 2402–2701, and antibodies for three specific IP_3_R isoforms (2699–2749 of IP_3_R-1, 2645–2695 of IP_3_R-2, and 2621–2671 IP_3_R-3) were obtained from Santa Cruz Biotechnology, Inc. Another anti-pan-IP_3_R antibody (mapping sequence is not told) was from Calbiochem (Merck KGaA Darmstadt, Germany). Anti-Cx43 (goat polyclonal IgG: mapping within the last 50 amino acids of human Cx43 origin; rabbit polyclonal IgG: mapping between amino acids 233–382 of Cx43 C-terminus of human origin), anti-S262 (250–300 of p-Cx43 of mouse protein), anti-S368 (around serine 368 of p-Cx43 of human protein) and anti-S279/282 phospho (250–300 of p-Cx43 of human protein), anti-α-actinin (amino acids 841–891 of α-actinin of human), anti-GAPDH (mouse monoclonal IgG1 FL of rabbit GAPDH) and anti-HA-probe (mapping within an internal region of the influenza HA protein) antibodies were from Santa Cruz Biotechnology, CA, USA. All the secondary antibodies and the reagents used were purchased from Abcam (Hong Kong) Ltd. or Sigma-Aldrich (St Louis, MO, USA) respectively. The fluorescent secondary antibody chicken-anti-goat and donkey-anti rabbit were obtained from Invitrogen Inc, Carlsbad, California, USA.

***Tissues***

Ventricular tissues were obtained from 12-week-old adult C57BL/6J mice and 1 to 2-day-old Sprague–Dawley rats. After mice were anesthetized with 10% chloralhydrate (0.1 ml/20 g, i.p), the hearts were immediately taken out and washed in Ca^2+^ and Mn^2+^-free Earle’s balanced salt solution. The ventricles were carefully separated from the hearts and were infiltrated with Tissue Tek OCT compound (SAKURA) and rapidly frozen in liquid nitrogen for 30 seconds. Serial sections (12 μm) were cut on a cryostat and collected on poly-D-lysine-coated slides for immunofluorescent studies.

***Isolation and culture of cardiomyocytes***

As described previously [2], adult ventricular myocytes were isolated by the method of retrogradely perfused whole heart in a Langendorff system with a modified HEPES buffered Tyrode solution that were equilibrated with O_2_ for at least 20 minutes before use. Cells from dissociated tissue were subjected to a progressive normalization of Ca^2+^ levels to a final concentration of 1 mM. Rod-shaped cells were selected by gently centrifuging (500 rpm, 1 minute) and plated in laminin-coated dishes with medium 199 containing 5% fetal calf serum. Two hours after plating in incubator, broken nonattached cells were discarded by gently washed two times with HEPES buffered Tyrode solution and end-to-end paired myocytes were chosen for the experiment.

Neonatal rat ventricular myocytes (NRVMs) were isolated from 1 to 2-day-old Sprague–Dawley rats by collegenase II digestion processes and further cultured for 48–72 hours before use as previously described [2,3]. HEK293 cells were obtained from the American Type Culture Collection. Both of the cells were cultured at 37 °C in Dulbecco's modified Eagles medium containing 10% fetal bovine serum and 2 mM glutamine in humidified 95% air and 5% CO_2_ incubator and harvested after passage for two times.

***Confocal Ca^2+^ imaging***

Cultured NRVMs were loaded with 4 μM fluo4/AM at 37 °C for 30 minutes and washed with HEPES-buffered salt solution (HEPES mM: NaCl 135, KCl 5, MgCl_2_ 1, CaCl_2_ 1.8, HEPES 10 and glucose 11, with pH 7.4 adjusted by NaOH) for 20 minutes as described previously [2,3]. Confocal images of fluo-4 fluorescence (excitation at 488 nm and emission detection at >515 nm) were obtained using Leica SP5 microscopy equipped with a 63× oil immersion objective (NA 1.4) at room temperature of 22–24 °C. [Ca^2+^]_i_ increases are presented as background-subtracted normalized fluorescence (*F*/*F_0_*).

***Gap-FRAP***

NRVMs and end-to-end paired adult mouse ventricular myocytes were loaded with the dye 6-CFDA (7 μg/ml), a specific dye known to permeate GJ after hydrolysis to 6-carboxyfluorescein [4–6] for 5 minutes at room temperature, and then were washed three times for 10 minutes. Fluorescence recovery after photobleach (FRAP) was measured by the laser scanning microscope (Leica SP5) equipped with 63× oil immersion objective. Briefly, a cell adjacent to other cells was selected and its fluorescence was photobleached by strong laser pulses (488 nm) with 30 scans for a total duration of 15 and 20 seconds, reaching almost complete and approximately 60% photobleaching in NRVMs and adult myocytes, respectively. The fluorescence intensity was recorded at excitation 488 nm and emission 570 nm in the bleached cells every 2–10 seconds over a period of 7 minutes before and after photobleaching. In each experiment, an isolated bleached cell and a labeled isolated cell, left unbleached, served as reference controls for the loss of fluorescence due to repeated scanning and dye leakage. After correction and normalization, fluorescence was plotted over time to generate fluorescence recovery curves. Analysis allows us to obtain two values by equations: the degree (%) and the rate of recovery.

**Preparation of shRNA for IP_3_R subtype**

Recombinant adenovirus for silencing *IP_3_R* was prepared with the BLOCK-iT Adenoviral RNAi Expression System (Invitrogen). The sequences of the oligonucleotides for *IP_3_R* RNA interference were as follows: IP_3_R-1: forward, CACCGCACTTGAACCAGATTATAGACGAATCTATAATCTGGTTCAAGTGC; reverse, AAAAGCACTTGAACCAGATTATAGATTCGTCTATAATCTGGTTCAAGTGC. IP_3_R-2: forward, CACCGGTACCAGCTAAACCTCTTTGCGAACAAAGAGGTTTAGCTGGTACC; reverse, AAAAGGTACCAGCTAAACCTCTTTGTTCGCAAAGAGGTTTAGCTGGTACC. IP_3_R-3: forward, CACCGCACATGAAGAGCAACAAATACGAATATTTGTTGCTCTTCATGTGC; reverse, AAAAGCACATGAAGAGCAACAAATATTCGTATTTGTTGCTCTTCATGTGC. Adenoviral vector containing a scrambled shRNA sequence at the same multiplicity of infection served as the control; forward, 5'-CACCGCCTGCCGTCCAAAGTTGTAACGAATTACAACTTTGGACGGCAGGC-3; reverse, 5'-AAAAGCCTGCCGTCCAAAGTTGTAATTCGTTACAACTTTGGACGGCAGGC-3. NRVMs were cultured for 24 hours, and then transduced with adenovirus carrying each IP_3_R isoform silencing gene (virus=30 m.o.i.) and scrambled gene (30 m.o.i.), respectively, and were further cultured for 48 hours. For knockdown of pan-IP_3_R, the three shRNA viruses were used in combination (30 m.o.i. for each). Significant reductions in each isoform of IP_3_R and pan-IP_3_R expressed in NRVMs were obtained (Supplementary Figure). ***Cx43 mutagenesis and infection***

The site-directed mutagenesis plasmids and adenovirus were constructed by Invitrogen Inc. The mutants include S279A, S282A, S282D and S279A/282A. The template was plasmid pIRES2-EGFP-Cx43 (constructed by Invitrogen) and the primers were:

S279A: Cx43-T835G-F: 5'-GCCTATGTCTCCTCCTGGGTACA-3' and Cx43-T835G-R: 5'-GCGAGTGGAGCCGTTGGTGA-3';

S282A: Cx43-T844G-F: 5'-TCTCGCCTATGGCTCCTCCTG-3' and Cx43-T844G-R: 5'-GTGGAGCCGTTGGTGAGGAG-3';

S279A/282A:Cx43-T835G, T844G-F:5'-TCACCAACGGCTCCACTCGCGCCTATGGCTCCTCCTGGGTACAAG-3' and Cx43-T835G, T844G-R: 5'-CTTGTACCCAGGAGGAGCCATAGGCGCGAGTGGAGCCGTTGGTGA-3'; S282D: Cx43-C845A-F: 5'-ACTCTCGCCTATGGATCCTCCTGGGTACAAGCTGGTTACTGG-3' and Cx43-C845A-R: 5'-TGTACCCAGGAGGATCCATAGGCGAGAGTGGAGCCGTTGGTG-3'.

The amino acids at the site of serine 279 (TCG), serine 282 (TCT) and serine 282 (TCT) are coded to alanine (GCG), alanine (GCT) and aspartic acid (TAT) in the Cx43 cDNA, respectively. The PCR experiment was performed as follow: denaturation at 95 °C for 30 seconds, 15 cycles of 30 seconds at 95 °C, 1 minute at 55 °C for annealing, 8 minutes at 72 °C for extension. At last the samples were maintained at 4 °C. The expansion product incubated in ice for 5 minutes, then at room temperature. Add 1 μl (10 U/μl) Mutazyme^TM^ Enzyme to the samples incubated for 1 hour at 37 °C. After reaction, 10 μl samples were transformed into 50 μl Escheichia coli DH5 α-cells. The mutations were analyzed by sequencing, and the correct one was selected. Recombinant plasmids and adenoviral vectors carrying wt-Cx43, S279A, S282A, S282D and S279A/282A genes were then constructed.

NRVMs were cultured for 24 hours before being transduced with wt-Cx43 (20 m.o.i.), mutants S279A, S282A, S282D, or S279A/282A (10, 2, 10 and 2 m.o.i.), respectively, and were further cultured for 24 hours before use. HEK293 cells were transfected with 2 μg/ml plasmids carrying rat wt-Cx43, S279A, S282A, and S279A/282A mutant for 48 hours before harvested.

***Co-immunoprecipitation***

NRVMs were lysed in cell lysis buffer (Cell Signaling Technology, Inc. Danvers, MA, USA) containing 2 mM polymethylsulfonylfluoide and complete protease inhibitor cocktail for 1 hour at 4 °C. The lysate was centrifuged at 14,000 × *g* at 4 °C for 10 minutes and the supernatant was used at once or stored at −80 °C until used. For immunoprecipitations, NRVMs lysates (1 mg) were incubated with 30 μl rProtein G Agarose (Invitrogen) for 3 hours at 4 °C and then centrifuged at 14,000 ×*g* at 4 °C for 10 minutes. The supernatant fraction were transferred to fresh tubes and added 10 μl normal rabbit or goat lgG as control group and 10 μl anti-IP_3_R-1, anti-IP_3_R-2, anti-IP_3_R-3, pan-IP_3_R or anti-Cx43 antibody, respectively. After incubated at 4 °C for 2 hours on a rotator and adsorbed onto 30 μl rProtein G Agarose overnight at 4 °C, the complexes immunoabsorbed to the beads were washed three times with ice-cold PBS, and the supernatant was diluted in sample buffer to be analyzed for Cx43 or IP_3_R expression by immunoblotting.

***Immunoblotting***

Lysates from NRVMs (30 μg to 80 μg) and the pulldown of IP_3_R or Cx43 were prepared and resolved on 8% and 10% SDS-PAGE gel for detection of IP_3_R or Cx43 expression, respectively. Membranes were blocked with 5% nonfat milk in Tris-buffered saline containing 0.1% (v/v) Tween 20 for 60 minutes at room temperature. Two commercially available antibodies (1:500 and 1:200), specific polyclonal antibodies for IP_3_R-1, IP_3_R-2, IP_3_R-3 (1:200, respectively), and antibodies for anti-Cx43 or for specific phosphor-amino acids of the carboxy-terminus of Cx43, including pS368, pS262 and pS279/282 (1:1000), were used overnight at 4 °C. The immunoblotted membrane was then incubated with horseradish peroxidase-conjugated secondary antibody for 1 hour and immunoreactive bands were detected by using enhanced chemiluminescence. The phosphorylation of each amino acid was quantified by correction and normalization against total Cx43 and actin or GAPDH immunoblotted by specific antibody.

The extraction of non-junctional and junctional protein lysates was performed as previously described [2]. GAPDH detection was used for confirmation of successful separation of junctional and non-junctional fractions.

***Immunocytochemistry and confocal imaging***

The immunocytochemistry of NRVMs, mouse and neonatal rat ventricle tissue were performed as previously described [2,3]. For co-staining assessment, NRVMs were fixed in 4% formaldehyde in phosphate-buffered saline (PBS) for 10 minutes and permeabilized with 0.1% triton X-100 for 10 minutes at room temperature. After blocking in PBS containing 5% bovine serum albumin for 1 hour, anti-Cx43 (1:100) and anti-pan-IP_3_R (1:100) was used overnight at 4 °C and for 1 hour at room temperature. The secondary antibodies Alexa Fluor 488-labeled donkey-anti-rabbit (1:500) and Alexa Fluor 594-labeled chicken-anti-goat (1:500) were applied for 1 hour at 37 °C. The nucleus was labeled with Hoechst 33258 at final concentration of 1 μg/ml. For co-staining of ventricle tissue sections, the procedure was similar as the co-staining assay in NRVMs, except for the fixed time in ventricle tissue changing to 2–4 hours, Slides were imaged in Z-series every 0.5 μm with Leica TCS SP5 inverted confocal microscope. The velocity software was applied to analyze the intercalated disc by creating three-dimension reconstructions. Tissue analysis was performed at equal magnifications over equivalent tissue areas and thickness. For the Cx43 labeling in NRVM and HEK293 cells, the protocol is the same as that in co-immunostaining assay, except for the anti-HA-probe antibody used was 1:500).

***Lucifer yellow uptake assay***

**A**s previously reported [7,8], LY can enter cell through gaps/hemichannels in cells, but dextran-rhodamine B cannot, thus, HEK293 cells and NRVMs grown to subconfluence on dishes were incubated in a Ca^2+^-containing HBSS. Cells were then exposed to HBSS containing 1 mM EGTA, 2% LY, and 0.5% 10 kDa dextran-rhodamine B for 8 minutes. After a brief wash with Ca^2+^-containing HBSS, LY (excitation at 405 nm and emission detection at 530 nm) and rhodamine B fluorescence (excitation at 570 nm and emission detection at 590 nm) from cells was imaged with the fluorescence microscope, respectively.

***Statistics***

Data were analyzed and presented as means ± standard deviation (SD) of *n* measurements. Statistical comparisons between groups were carried out with 2-way unpaired Student’s *t-*test following analysis of variance (ANOVA). The accepted level of significance was *P* < 0.05.

**References**

1. Li W, Schultz C, Llopis J, Tsien RY: **Membrane-permeant esters of inositol polyphosphates, chemical syntheses and biological applications**. *Tetrahedron* 1997, **53**:12017–12040.

2. Li C, Meng Q, Yu X, Jing X, Xu P, Luo D: **Regulatory effect of connexin 43 on basal Ca^2+^ signaling in rat ventricular myocytes**. *PLoS ONE* 2012, **7**:361–365.

3. Luo D, Yang D, Lan X, Li K, Li X, Chen J, Zhang Y, Xiao RP, Han Q, Cheng H: **Nuclear Ca^2+^ sparks and waves mediated by inositol 1,4,5-trisphosphate receptors in neonatal rat cardiomyocytes**. *Cell Calcium* 2008, **43**:165–174.

4. Wade MH, Trosko JE, Schindler M: **A fluorescence photobleaching assay of gap junction-mediated communication between human cells**. *Science* 1986, **232**:525–528.

5. Santiquet NW, Develle Y, Laroche A, Robert C, Richard FJ: **Regulation of gap-junctional communication between cumulus cells during in vitro maturation in swine, a gap-FRAP study**. *Biol Reproduction* 2012, **87**:1–8.

6. Matsushita S, Kurihara H, Watanabe M, Okada T, Sakai T, Amano A: **Alterations of phosphorylation state of connexin 43 during hypoxia and reoxygenation are associated with cardiac function**. *J Histochem Cytochem* 2006, **54**:343–353.

7. Doble BW, Chen Y, Bosc DG, Litchfield DW, Kardami E: **Fibroblast growth factor-2 decreases metabolic coupling and stimulates phosphorylation as well as masking of connexin43 epitopes in cardiac myocytes**. *Circ Res* 1996, **79**:647–658.

8. Opsahl H, Rivedal E: **Quantitative determination of gap junction intercellular communication by scrape loading and image analysis**. *Cell Adhes Commun* 2000, **7**:367–375.
